# GmTOC1b inhibits nodulation by repressing *GmNIN2a* and *GmENOD40-1* in soybean

**DOI:** 10.3389/fpls.2022.1052017

**Published:** 2022-11-11

**Authors:** Yuhang Zhang, Qun Cheng, Chunmei Liao, Lanxin Li, Chuanjie Gou, Zheng Chen, Yanan Wang, Baohui Liu, Fanjiang Kong, Liyu Chen

**Affiliations:** Guangdong Key Laboratory of Plant Adaptation and Molecular Design, Guangzhou Key Laboratory of Crop Gene Editing, Innovative Center of Molecular Genetics and Evolution, School of Life Sciences, Guangzhou University, Guangzhou Higher Education Mega Center, Guangzhou, China

**Keywords:** soybean, nodulation, *TOC1*, *NIN*, *ENOD40*

## Abstract

Symbiotic nitrogen fixation is an important factor affecting the yield and quality of leguminous crops. Nodulation is regulated by a complex network comprising several transcription factors. Here, we functionally characterized the role of a TOC1 family member, GmTOC1b, in soybean (*Glycine max*) nodulation. RT-qPCR assays showed that *GmTOC1b* is constitutively expressed in soybean. However, *GmTOC1b* was also highly expressed in nodules, and GmTOC1 localized to the cell nucleus, based on transient transformation in *Nicotiana benthamiana* leaves. Homozygous *Gmtoc1b* mutant plants exhibited increased root hair curling and produced more infection threads, resulting in more nodules and greater nodule fresh weight. By contrast, *GmTOC1b* overexpression inhibited nodulation. Furthermore, we also showed that GmTOC1b represses the expression of nodulation-related genes including *GmNIN2a* and *GmENOD40-1* by binding to their promoters. We conclude that GmTOC1b functions as a transcriptional repressor to inhibit nodulation by repressing the expression of key nodulation-related genes including *GmNIN2a*, *GmNIN2b*, and *GmENOD40-1* in soybean.

## Introduction

Soybean (*Glycine max* L.) is an important oil crop and the main source of protein for animal diets. To meet their high nitrogen demand during growth and reproduction, legumes release flavonoids *via* root exudates to attract rhizobia with which they then form nodules (Hungria et al., 2005; Ciampitti and Salvagiotti, 2018). Nodules function as a biological nitrogen fixation factory, which can efficiently convert atmospheric N_2_ into ammonia for their plant hosts (Schipanski et al., 2010; Dong et al., 2021b). In exchange, legumes can provide a stable nitrogen fixation environment that is low in oxygen, as well as carbon and other nutrients for rhizobia. Thus, symbiotic nitrogen fixation not only is a critical factor for improving soybean yield and quality but can also help us reach and maintain sustainable agricultural production practices in an environmentally conscious manner (Gelfand and Philip Robertson, 2015; Santachiara et al., 2019).

The symbiosis between legumes and rhizobia has been well studied. Rhizobia are attracted to plant roots by host-secreted flavonoids and synthesize nodulation (Nod) factors, which are specifically recognized by NOD FACTOR RECEPTOR (NFR) on the root epidermis of legumes (Indrasumunar et al., 2010; Indrasumunar et al., 2011; Liu and Murray, 2016). After initial signal recognition, root hairs curl to surround the rhizobia, followed by the entry of the rhizobia into root endodermis cells through infection lines (Suzaki and Kawaguchi, 2014; Kawaharada et al., 2017). The invaded root cortical cells divide to form a nodule primordium (Heckmann et al., 2011). The rhizobia are finally released into each primordium through cortical cells to develop a mature root nodule (Hayashi et al., 2014).

Many key genes involved in symbiotic nitrogen fixation have been identified. After recognizing Nod factors, NFRs can induce the expression of genes encoding leucine-rich repeat receptor-like kinases (LRR-RLKs) such as *Symbiosis receptor kinase* (*LjSYMRK*) from *Lotus japonicus* (Demchenko et al., 2004), *DOES NOT MAKE INFECTION 2* (*MtDMI2*) from *Medicago truncatula* (Limpens et al., 2005), and *Nodulation receptor kinase* (*GmNORK*) from soybean (Indrasumunar et al., 2015; Wang et al., 2020b), which leads to the activation of downstream gene *DMI3*, encoding the intracellular calcium and calmodulin-dependent protein kinase (CCaMK) (Rival et al., 2012). CCaMK can phosphorylate the transcription factor CYCLOPS and then activate the expression of its downstream target gene *NIN* (*Nodule induction*) (Cerri et al., 2017; Liu et al., 2019). *EARLY NODULIN 40* (*ENOD40*) was the first identified key component of nodulation and encodes a host-derived 12- to 13-amino acid peptide that enhances the stability of sucrose synthase (Crespi et al., 1994; Röhrig et al., 2002). Previous research suggested that *ENOD40* expression can be rapidly induced after inoculation with rhizobia or purified Nod factors, with ENOD40 functioning as an intercellular signal molecule or participating in the regulation of carbon metabolism during nodule formation (Campalans et al., 2004; Xu et al., 2021). In addition, the transcription factors Nodulation Signaling Pathway 1 (NSP1) and NSP2 form a heterologous protein complex in the nucleus (Smit et al., 2005) and directly bind to the promoters of early nodulin genes such as *ENOD11* (Svistoonoff et al., 2010), *NIN* and *Ethylene response factor required for nodule* (*ERN*) (Cerri et al., 2012), which regulate nodule formation at the early stage. Although many studies have investigated nodule signaling, a full picture of nodulation has yet to emerge due to the underlying complex regulatory pathways.

TOC1 (TIMING OF CAB EXPRESSION 1) is a key component of the circadian clock and controls many biological processes supporting plant growth and development (Sanchez et al., 2011; Scheiermann et al., 2013; Xu et al., 2022). In *Arabidopsis* (*Arabidopsis thaliana*), *toc1* mutants enhance drought stress tolerance and showed greater sensitivity to the phytohormone abscisic acid (ABA). Moreover, TOC1 was shown to bind to the promoter of the *ABA receptor* (*ABAR*) and modulate its expression (Legnaioli et al., 2009). TOC1 also interacts with PHYTOCHROME-INTERACTING FACTOR 4 (PIF4) and represses its ability to suppress thermoresponsive growth in the evening (Zhu et al., 2016). In rice (*Oryza sativa*), OsTOC1 regulates tiller-bud and panicle development by indirectly repressing the expression of *TEOSINTE BRANCHED 1* (*TB1*), *DWARF 14* (*D14*), and *IDEAL PLANT ARCHITECTURE 1* (*IPA1*) (Wang et al., 2020a). In the model legume *M. truncatula*, members of other clock components, including LATE ELONGATED HYPOCOTYL (LHY) and LUX ARRHYTHMO (LUX), also regulate nodulation. For instance, MtLHY affected nodulation *via* the regulation of nodule cysteine-rich peptides (Kong et al., 2020; Achom et al., 2021). MtLUX also plays a role in nodule formation, probably through an indirect regulation with MtLHY (Kong et al., 2022).

In this study, we characterized the role of *GmTOC1b* in nodule formation. Knockout mutants of *GmTOC1b* promoted soybean nodulation by affecting hair curling and the number of infection threads. Conversely, overexpression of *GmTOC1b* significantly inhibited nodulation in transgenic hairy roots. *GmTOC1b* regulated multiple nodulation-related genes, including *GmNIN2a*, *GmNIN2b*, and *GmENOD40-1*, in the early stage. Further investigation showed that GmTOC1b represses the expression of *GmNIN2a* and *GmENOD40-1* by binding to their promoters. Taken together, our results revealed the vital role of GmTOC1b in regulating nodule formation in soybean and should be useful for genetic improvement in soybean and other legumes.

## Materials and methods

### Plant materials and growth conditions

Soybean (*G. max* L.) cultivar Williams 82 (W82) and GUS-tagged *Bradyrhizobium japonicum* strain USDA110 were used in this study. All soybean plant materials were generated in the W82 background.

For tissue-specific expression analysis, soybean seeds were sown and cultured at 26°C under 16-h-light/8-h-dark conditions. Tissue samples were harvested at the indicated growth periods. All samples were frozen in liquid nitrogen and stored at −80°C until total RNA extraction.

For the nodulation phenotyping assay, uniform healthy seeds were surface sterilized with chlorine gas for 14 h and then germinated in vermiculite under 16-h-light/8-h-dark conditions in a growth room at 26°C. Soybean seedlings were supplied with low-nitrogen nutrient solution containing 150 μM of KNO_3_, 120 μM of Ca(NO_3_)_2_·4H_2_O, 25 μM of MgCl_2_, 30 μM of (NH_4_)_2_SO_4_, 40 µM of Fe-Na-EDTA, 500 μM of MgSO_4_·7H_2_O, 1.5 μM of MnSO_4_·H_2_O, 1.5 μM of ZnSO_4_·7H_2_O, 0.5 μM of CuSO_4_·5 H_2_O, 0.15 μM of (NH_4_)_6_Mo_7_O_24_·4H_2_O, 2.5 μM of NaB_4_O_7_·10H_2_O, 500 μM of KH_2_PO_4_, 540 μM of CaCl_2_, and 345 μM of K_2_SO_4_. After unifoliolate leaves were completely opened, the seedlings were inoculated with GUS-tagged *B. japonicum* USDA110 and resuspended in low-nitrogen nutrient solution (OD_600_ = 0.1, 5 ml) at Zeitgeber 12. The nodule phenotype was evaluated 14 days after inoculation (DAI).

### Phylogenetic analysis

TOC1 homologous protein sequences from monocotyledons and dicotyledons were aligned using the Clustal X. A neighbor-joining tree was constructed with the software MEGA11 (Tamura et al., 2021). Conserved motifs were extracted with the MEME suite (Bailey et al., 2015).

### RNA extraction and RT-qPCR analysis

Total RNA was extracted using a FastPure Plant Total RNA Isolation Kit (Vazyme, Nanjing, China). Removal of genomic DNA and first-strand cDNA synthesis was performed using a PrimeScript RT reagent kit with gDNA Eraser (Takara, Dalian, China). qPCR was conducted on a Roche LightCycle480 system (Roche, Mannheim, Germany) with TB Green (Takara). Three biological replicates were used for all experiments. *GmActin* (*Glyma.02g091900*) was used as the reference gene. All primers used are listed in **Supplementary Table 1**.

### Plasmid construction

CRISPR/Cas9-mediated gene editing was used to generate *Gmtoc1b* mutants. Single-guide RNAs (sgRNA) were predicted using the online tool CRISPR-P 2.0 (http://crispr.hzau.edu.cn/cgi-bin/CRISPR2/CRISPR); the two most reliable sgRNAs targeting different regions of *GmTOC1b* were designed and cloned into the CRISPR/Cas9 vector (Hao et al., 2020). The construct was transformed into *Agrobacterium* (*Agrobacterium tumefaciens*) strain EHA105, which was then used to transform soybean cultivar W82 as previously described (Chen et al., 2018). The genotype of *Gmtoc1b* mutants was verified using Sanger sequencing, and homozygous *Gmtoc1b* mutants were used for further research. For the *GmTOC1b* overexpression construct, the *GmTOC1b* coding sequence without stop codon was amplified and cloned into the *proGmUBI:3×Flag* vector under the control of the soybean *Ubiquitin* (*UBI*) promoter to generate *proGmUBI : GmTOC1b-3×Flag*. The cauliflower mosaic virus (CaMV) 35S promoter was used to drive the expression of green fluorescent protein (*GFP*) for screening positive transformation events.

### Subcellular localization of GmTOC1b

The *GmTOC1b* coding sequence without the stop codon was cloned in-frame and upstream of the *GFP* sequence driven by the *GmUBI* promoter. The plasmid was transformed into *Agrobacterium* strain GV3101, which was then transiently infiltrated into *Nicotiana benthamiana* leaves. The plants were grown at 26°C under 16-h-light/8-h-dark conditions for 48 h. Green fluorescent protein (GFP) fluorescence was observed using a Zeiss LSM 800 confocal laser scanning microscope (Zeiss, Oberkochen, Germany). Transiently infiltrated *proGmUBI : GFP N. benthamiana* leaves were used as localization controls. Leaves were stained with 4′,6-diamidino-2-phenylindole (DAPI) to label nuclei.

### Soybean hairy root transformation

To obtain transgenic composite plants, soybean hairy root transformation was performed according to a previously published method (Kereszt et al., 2007). Positive transgenic hairy roots were confirmed by detecting GFP fluorescence using a hand-held fluorescence lamp (Luyor 3415RG; Luyor, Shanghai, China). The plants were grown under high-humidity conditions for 1 week to adapt to the environment. For the nodulation phenotyping assay, each plant was inoculated with *B. japonicum* USDA110 (OD_600_ = 0.1, 5 ml). Nodule numbers per root and shoot dry weights were evaluated at 20 DAI.

### Observation of root hair curling and infection threads

To detect infection events, 7-day-old seedlings were inoculated with GUS-tagged *B. japonicum* USDA110. Lateral root segments (5 cm in length) from W82 and the *Gmtoc1b* mutants were harvested at 3 and 5 DAI. For the detection of root hair curling, root samples of 3-DAI seedlings were gently washed with sterile water and directly used for observation. For detection of infection threads, 5-DAI root samples were washed and then soaked in X-Gluc solution [100 mM of NaH_2_PO_4_, 100 mM of Na_2_HPO_4_, 1 mM of K_4_(Fe(CN)_6_), 1 mM of K_3_(Fe(CN)_6_), 1 mg/ml of X-Gluc, 10 mM of Na_2_EDTA, 0.1% (v/v) Triton X-100] overnight at 37°C, after which time the stained roots were observed. Digital images were taken with a Zeiss Axio Imager A2 microscope (Zeiss, Oberkochen, Germany).

### Transient expression assay

To generate promoter-driven firefly luciferase (*LUC*) constructs, ~2-kb promoter fragments for *NIN2a* and *ENOD40-1* were individually amplified from W82 genomic DNA; the PCR product was purified and cloned into the pGreenII 0800-LUC vector. The resulting *proGmNIN2a:LUC* and *proGmENOD40-1:LUC* constructs were used as reporters, and *proGmUBI:3×Flag* and *proGmUBI : GmTOC1b-3×Flag* were used as effectors. The constructs *proGmUBI : GmTOC1b-3×Flag* and *proGmNIN2a:LUC* or *proGmENOD40-1:LUC* were co-infiltrated into *N. benthamiana* leaves. The constructs *proGmUBI:3×Flag* and *proGmNIN2a-LUC* or *proGmENOD40-1-LUC* were co-infiltrated into *N. benthamiana* leaves as the blank control. Relative Firefly Luciferase; (LUC)/Renilla Luciferase (REN) was analyzed. Three independent biological replicates were performed in this assay. The firefly and Renilla luciferase signals were assayed using a Dual-Luciferase Reporter Assay System (Promega, Madison, WI, USA) and measured on a Biotek Synergy H1 Microplate Reader (Agilent, Santa Clara, CA, USA). Digital images were taken after spraying 1 mM of d-Luciferin sodium salt onto infiltrated *N. benthamiana* leaves (Sangon Biotech, Shanghai, China).

## Results

### Sequence analysis of GmTOC1b

**Figure 1:**
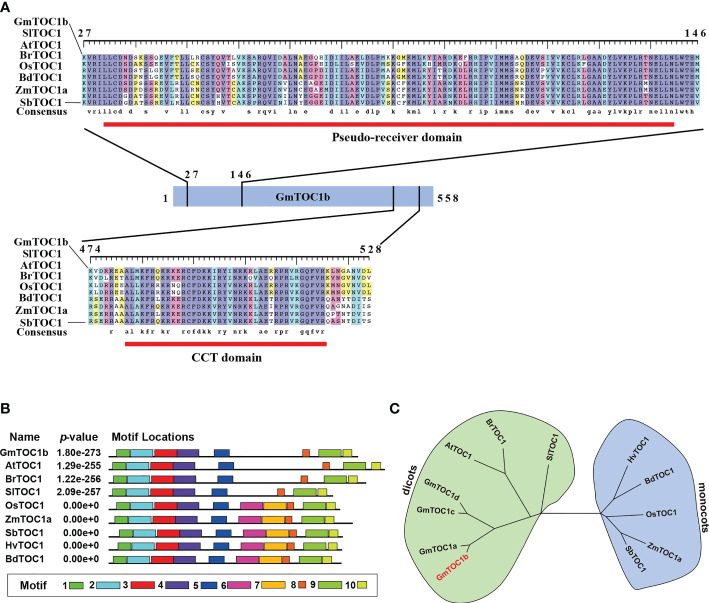
Analysis of TOC1 homologs in soybean and other plants. **(A)** TOC1 homologs contain conserved an N-terminal pseudo-receiver domain and a C-terminal CCT (CONSTANS, CO-LIKE, and TOC1) domain. Amino acid sequences were aligned using Clustal X. Red lines indicates the highly conserved domains. aa, amino acids; Sl, *Solanum lycopersicum*; At, *Arabidopsis thaliana*; Br, *Brassica rapa*; Os, *Oryza sativa*; Bd, *Brachypodium distachyon*; Zm, *Zea mays*; Sb, *Sorghum bicolor*. **(B)** Distribution of conserved motifs in TOC1 homologs. The MEME website tool was used to perform the analysis. **(C)** Phylogenetic analysis of TOC1 proteins. A neighbor-joining tree was constructed using full-length protein sequences, with bootstrap values set to 1,000 replicates.

In this study, we characterized the soybean circadian regulator gene *GmTOC1b* (also known as *PSEUDO-RESPONSE REGULATOR 1* [*PRR1*]), showing high similarity to *Arabidopsis TOC1* (Gan et al., 2020). We downloaded the *GmTOC1b* (*Glyma.06G196200*) sequence from the Phytozome (https://phytozome-next.jgi.doe.gov) database. The cDNA sequence of *GmTOC1b* is 2,597 bp with an open reading frame consisting of 1,677 bp that encodes a 559-amino acid protein. GmTOC1b is predicted to contain an N-terminal pseudoreceiver (PR) domain and a C-terminal [CONSTANS, CONSTANS-LIKE, TOC1] (CCT) domain, which are common to all TOC1 orthologs (**Figure 1A**). Phylogenetic analysis indicated that GmTOC1b groups into the dicots and is closely related to its soybean TOC1 homologs and other TOC1 proteins from *Arabidopsis*, *Brassica rapa*, and *Solanum lycopersicum* (**Figure 1C**). We also looked for conserved motifs across TOC1 homologs, which identified the PR (motifs 1, 2, and 3) and CCT (motif 9) domains in all TOC1 proteins (Wang et al., 2020a). Notably, motifs 5 and 8 only existed in the TOC1 proteins from monocots (**Figure 1B**). These results suggest that TOC1 is relatively well conserved with unique characteristics that have arisen since monocotyledons and dicotyledons diverged.

### GmTOC1b is a nuclear protein

**Figure 2:**
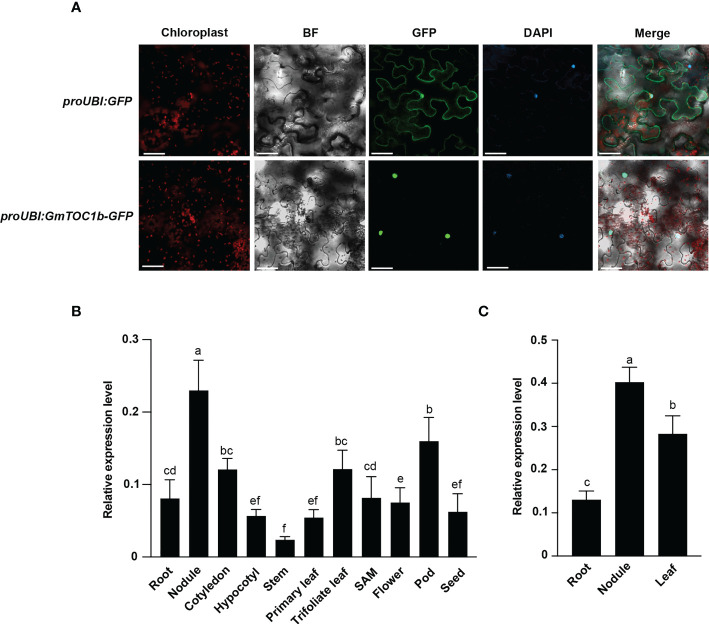
GmTOC1b localization and expression pattern analyses of *GmTOC1b*. **(A)** Subcellular localization of GmTOC1b. *Nicotiana benthamiana* leaves were transiently infiltrated with *proUBI : GFP* or *proUBI : GmTOC1b-GFP*; the plants were then grown for 48 h under 16-h-light/8-h-dark condition before GFP fluorescence was observed by microscopy. GFP, green fluorescent protein; BF, brightfield; Merge, GFP and BF images. Scale bars = 20 μm. **(B)** Relative *GmTOC1b* transcript levels in various tissues during growth and at different development stages. Relative expression level was calculated as the ratio of the expression value of the target gene to that of reference gene *GmActin* as an internal standard. **(C)** RT-qPCR analysis of *GmTOC1b* in different tissues at 14 days after inoculation (DAI) with rhizobia. Relative expression level was calculated as the ratio of the expression value of the target gene to that of reference gene *GmActin* as an internal standard. Three biological replicates were performed in all experiments. Different lowercase letters indicate significant differences as determined by Student’s *t*-test.

The subcellular distribution of GmTOC1b is essential for the prediction of its function. *GmTOC1b*-*GFP* driven by the *GmUBI* promoter was detectable specifically in the nucleus of *N. benthamiana* mesophyll cells (**Figure 2A**), whereas the GFP signal was distributed ubiquitously among the *N. benthamiana* mesophyll cells in the *proGmUBI*-GFP control. The results showed that GmTOC1b is a nucleus-localized protein.

### Expression patterns of *GmTOC1b* in soybean

We harvested samples from various tissues such as roots, nodules, cotyledons, hypocotyls, stems, primary leaves, trifoliate leaves, the shoot apical meristem (SAM), flowers, pods, and seeds 12 h after lights on (Zeitgeber 12 [ZT12]) from seedlings or plants grown under a 16-h-light/8-h-dark photoperiod. We detected *GmTOC1b* transcripts in all tissues tested, but with the highest abundance in nodules (**Figure 2B**). Furthermore, we also assessed *GmTOC1b* expression levels in leaves, roots, and nodules of soybean plants 14 days after inoculation (DAI) with *B. japonicum* strain USDA110. We determined that *GmTOC1b* is also highly expressed in nodules (**Figure 2C**), suggesting that GmTOC1b may function in the formation and development of nodules.

### Knockout of *GmTOC1b* promotes soybean nodulation

**Figure 3:**
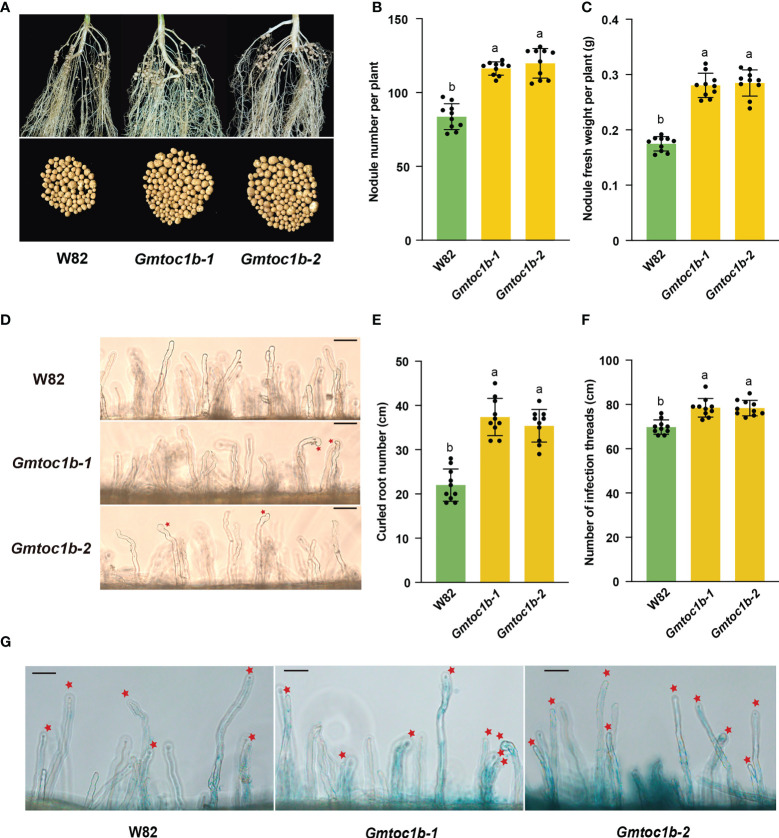
Knockout of *GmTOC1b* promotes nodulation in soybean. **(A)** Representative nodule performance of W82 and *Gmtoc1b* mutants at 14 DAI. **(B, C)** Nodule number and nodule fresh weight per plant for W82 and *Gmtoc1b* mutants at 14 DAI. Values are the mean ± SD. **(D)** Representative image of root hair curling in W82 and the *Gmtoc1b* mutant at 3 DAI. Scale bar, 100 μm. **(E)** Number of curled root hairs on W82 and *Gmtoc1b* mutant plants per centimeter of root length (n = 10). **(F)** Number of infection threads in W82 and *Gmtoc1b* mutants per centimeter of root length (n = 10). **(G)** Representative image of infection numbers observed in the W82 and *Gmtoc1b* mutants at 5 DAI (n = 10). Scale bars, 100 μm. Different lowercase letters indicate significant differences as determined by Student’s *t*-test.

To investigate a possible role for GmTOC1b in nodulation, we used clustered regularly interspaced short palindromic repeats (CRISPR)/CRISPR-associated nuclease 9 (Cas9)-mediated gene editing to generate *Gmtoc1b* mutants. We generated three positive transgenic events from 10 T0 generation transgenic plants. After the screening of heterozygous *Gmtoc1b* mutant lines, we obtained two homozygous T_2_ generations *Gmtoc1b* mutants with a 7-bp deletion (*Gmtoc1b-1*) or a 4-bp deletion (*Gmtoc1b-2*) (**Supplementary Figure 1A**). The T_3_ generation of homozygous *Gmtoc1b* mutants was used for further study. Reverse transcription–quantitative PCR (RT-qPCR) analysis showed that *GmTOC1b* expression levels are markedly lower in both *Gmtoc1b* mutants compared to W82 (**Supplementary Figure 1B**). These two mutants allowed us to investigate GmTOC1b function by evaluating the number of nodules per plant for the *Gmtoc1b* mutants and the wild-type W82 after inoculation with β-glucuronidase (GUS)-tagged *B. japonicum* USDA110. We observed a significant increase in nodule number and nodule fresh weight in the two *Gmtoc1b* mutants relative to W82 at 14 DAI (**Figures 3A–C**). As leghemoglobin plays a key role in the biological nitrogen fixation of leguminous plants (Berger et al., 2020; Du et al., 2020; Jiang et al., 2021), we further measured the content of leghemoglobin. The results showed that no significant difference existed between nodules from W82 and *Gmtoc1b* mutants (**Supplementary Figure 3**). The results indicated that the increased nodule number may not due to the functional weakening of nodules in *Gmtoc1b* mutants.

### Knocking out *GmTOC1b* enhances rhizobial infection and nodule formation

To explore the roles of GmTOC1b during rhizobial infection and nodule formation, we observed root phenotypes after inoculation. Compared to W82, the two *Gmtoc1b* mutants both exhibited significantly greater root hair curling at 3 DAI and more infection threads at 5 DAI (**Figures 3D–G**). Both *Gmtoc1b* mutants also produced more small nodules at 7 DAI relative to W82 (**Supplementary Figures 2A, B**).

### Overexpression of *GmTOC1b* inhibits nodulation

**Figure 4:**
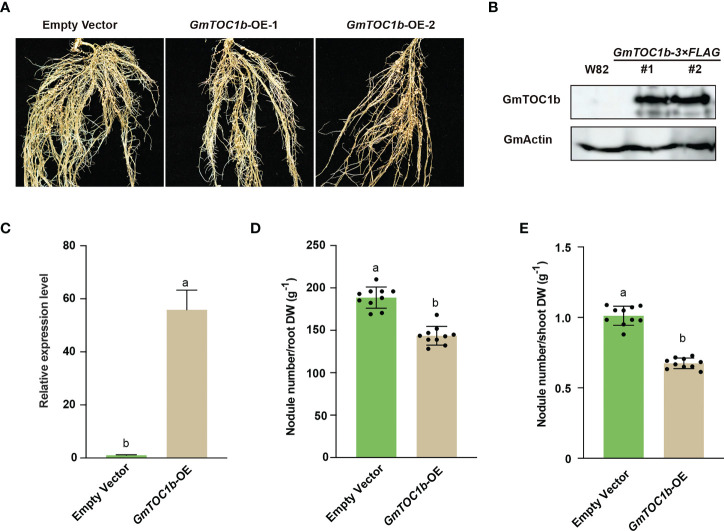
Overexpression of *GmTOC1b* in transgenic hairy roots inhibits nodulation. **(A)** Representative nodulation phenotype of *GmTOC1b-*OE and empty vector control transgenic hairy roots at 14 DAI. **(B)** Immunoblot analysis of GmTOC1b abundance in W82 and *GmTOC1b-3Flag* transgenic hairy roots. An anti-Flag antibody was used to recognize GmTOC1b-3Flag; actin was used as loading control. **(C)** Relative *GmTOC1b* transcript levels in *GmTOC1b-*OE and empty vector control transgenic hairy roots. Relative expression level was calculated as the ratio of the expression value of the target gene to that of reference gene *GmActin* as an internal standard. **(D, E)** Nodule number per gram of root dry weight or shoot dry weight in *GmTOC1b-*OE and empty vector control transgenic hairy roots at 14 DAI (n = 10). Different lowercase letters indicate significant differences as determined by Student’s *t*-test.

In a complementary approach, we also generated transgenic composite plants with hairy roots overexpressing *GmTOC1b* under the control of the soybean *Ubiquitin* (*GmUBI*) promoter, which we then inoculated with rhizobia. Relative *GmTOC1b* transcript levels were close to 60-fold higher in the overexpression (*GmTOC1b-*OE) lines; moreover, we detected Flag-tagged GmTOC1b, but not in empty vector control transgenic roots (**Figures 4B, C**). The transgenic *GmTOC1b*-OE roots formed fewer nodules compared to empty vector control hair roots at 20 DAI (**Figure 4A**). The lower number of nodules per gram of root or shoot dry weight also suggested that overexpression of *GmTOC1b* significantly inhibits nodulation (**Figures 4D, E**). Together, these data reveal that GmTOC1b plays a negative role in soybean nodulation.

### *GmTOC1b* inhibits the transcription of nodulation-related genes

**Figure 5:**
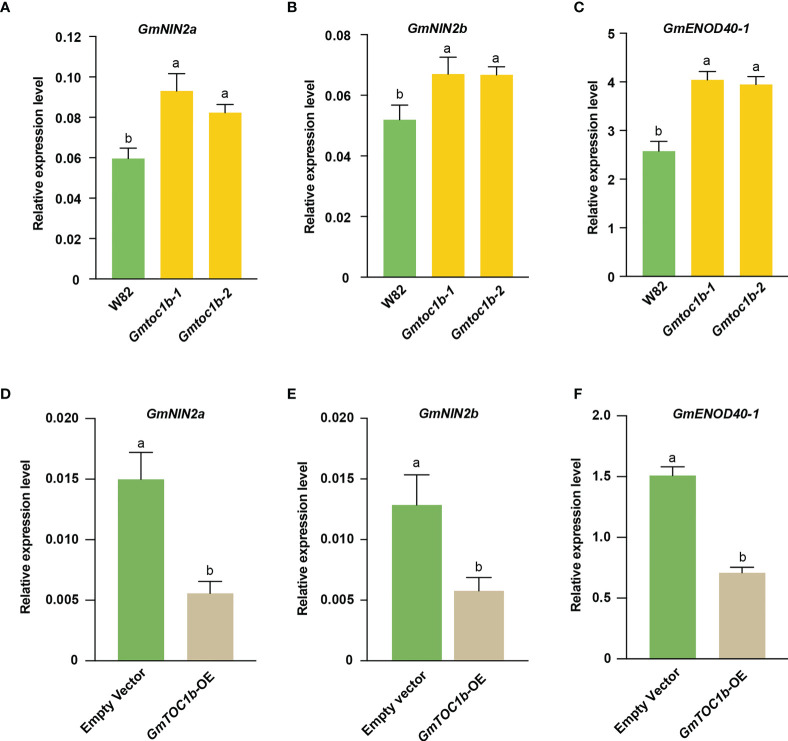
Expression of key nodulation-related genes in the early stage of rhizobia inoculation. Roots were harvested for analysis at 3 DAI. **(A–C)** Relative transcript levels of *GmNIN2a*, *GmNIN2b*, and *GmENOD40-1* in the roots of W82 and *Gmtoc1b* mutants. **(D–F)** Relative transcript levels of *GmNIN2a*, *GmNIN2b*, and *GmENOD40-1* in the roots of *GmTOC1b-*OE and empty vector control transgenic hairy roots. Relative expression level was calculated as the ratio of the expression value of the target gene to that of reference gene *GmActin* as an internal standard. Three biological replicates were performed in all experiments. Different lowercase letters indicate significant differences as determined by Student’s *t*-test.

To explore the molecular mechanisms by which GmTOC1b inhibits nodule formation, we analyzed the expression pattern of the nodulation-related marker genes *GmNIN2a*, *GmNIN2b*, and *GmENOD40-1* in infected soybean roots at 3 DAI. We first assessed their expression levels in the *Gmtoc1b* mutants, and all three genes were induced in the *Gmtoc1b* mutants (**Figures 5A–C**). By contrast, these three genes were expressed at much lower levels in transgenic hairy roots overexpressing *GmTOC1b* (**Figures 5D–F**). These results suggest that GmTOC1b functions as a negative regulator of these nodulation-related genes.

### GmTOC1b inhibits *GmNIN2a* and *GmENOD40-1* transcription *via* binding to their promoters

**Figure 6:**
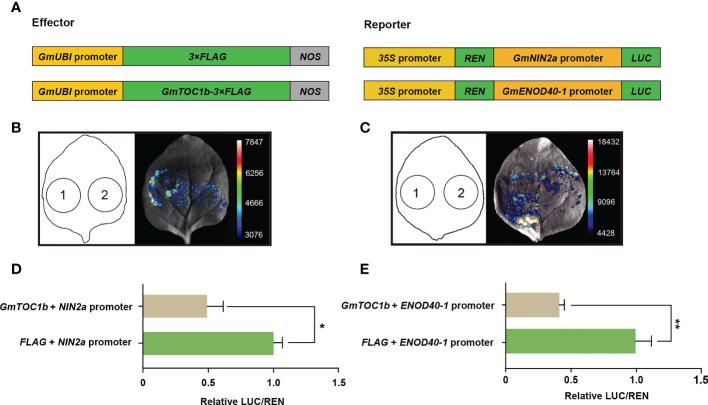
GmTOC1b represses the transcription of *GmNIN2a* and *GmENOD40-1*. **(A)** Schematic diagram of the constructs used for the transient co-transfection assay. **(B)** Representative image of firefly luciferase (LUC) activity driven by the *GmNIN2a* promoter. 1, *proGmUBI:3×Flag* + *proGmNIN2a:LUC*; 2, *proGmUBI : GmTOC1b-3×Flag* + *proGmNIN2a:LUC*. **(C)** Representative image of luciferase activity driven by the *GmENOD40-1* promoter. 1, *proGmUBI:3×Flag* + *proGmENOD40-1:LUC*; 2, *proGmUBI : GmTOC1b-3×Flag* + *proGmENOD40-1:LUC*. **(D, E)** GmTOC1b represses *GmNIN2a* and *GmENOD40-1* promoter activities. Relative firefly luciferase activity driven by the *GmNIN2a* **(D)** or *GmENOD40-1* **(E)** promoter was normalized to CaMV 35S promoter-driven *Renilla* luciferase (REN) activity. Data are means ± SD of seven independent samples (*p < 0.05; **p < 0.01).

Previous research showed that *Arabidopsis* TOC1 functions as a general transcriptional repressor (Gendron et al., 2012). Based on the lower expression of *GmNIN2a*, *GmNIN2b*, and *GmENOD40-1* in transgenic hairy roots overexpressing *GmTOC1b*, we speculated that GmTOC1b may bind to the promoters of these genes during nodulation. To test this hypothesis, we first analyzed 2-kb fragments of the *GmNIN2a*, *GmNIN2b*, and *GmENOD40-1* promoters to look for *cis*-elements bound by TOC1. We identified two typical HUD motifs in the *GmNIN2a* promoter (Michael et al., 2008a), one typical morning element motif, and three typical HUD motifs in the *GmENOD40-1* promoter (Michael et al., 2008b), suggesting that GmTOC1b may bind to these promoters. We then performed a dual-luciferase transient expression assay using the *GmNIN2a* and *GmENOD40-1* promoters individually driving the firefly luciferase (*LUC*) reporter gene together with an effector construct harboring *GmTOC1b* expressed from the soybean *Ubiquitin* (*GmUBI*) promoter (*proGmUBI : GmTOC1B-3Flag*) (**Figure 6A**). Co-infiltration of *N. benthamiana* leaves with the effector construct and each LUC reporter construct established that the presence of GmTOC1b significantly inhibits the LUC activity derived from the *GmNIN2a* and *GmENOD40-1* promoters compared to the empty effector vector control (*proGmUBI:3Flag*). Together, these results indicate that GmTOC1b represses the transcription of *GmNIN2a* and *GmENOD40-1*, likely by binding to their promoters (**Figures 6B–E**).

## Discussion

Symbiosis-mediated nitrogen fixation provides a large amount of nitrogen during soybean growth and reproduction (Pagano and Miransari, 2016). Therefore, the effect of nitrogen fixation is particularly important to soybean yield and quality. Importantly, the growth and proliferation of rhizobia require host-derived carbohydrates to support optimal utilization of nutrients, driving the co-evolution of a complex and precise regulation network between legumes and their rhizobial symbionts to modulate nodule number. Over the past 10 years, many key regulators of nodule formation have been reported (Kaló et al., 2005; Smit et al., 2005; Lim et al., 2011; Gamas et al., 2017; Suzaki and Nishida, 2019; Wang et al., 2019; Gao et al., 2021; He et al., 2021; Zhang et al., 2021). In soybean, components of the circadian clock, such as *EARLY FLOWERING 3* (*GmELF3*), *LATE ELONGATED HYPOCOTYL* (*GmLHY*), *GmPRR3*, and *LUX ARRHYTHMO* (*GmLUX*), were shown to be involved in the regulation of growth and development or abiotic stress tolerance (Lu et al., 2017; Cheng et al., 2020; Lu et al., 2020; Bu et al., 2021; Dong et al., 2021a; Fang et al., 2021; Li et al., 2021; Wang et al., 2021a). Members of the clock components including LHY and LUX play roles in regulating nodulation in the model legume *M. truncatula*. However, an effect of the circadian clock on nodulation has not previously been reported in soybean. Our results indicated that *GmTOC1b* was highly expressed in nodules. Furthermore, GmTOC1b inhibited soybean nodulation by repressing the transcription of the key nodulation-related genes *GmNIN2a*, *GmNIN2b*, and *GmENOD40-1*, demonstrating that GmTOC1 is a transcriptional repressor.

GmTOC1b is predicted to contain the typical N-terminal PR domain and C-terminal CCT domain of pseudo-response regulators. Previous research indicated that TOC1 directly binds to *cis*-elements located in the promoters of target genes through its CCT domain. Indeed, transient overexpression of *TOC1* in *Arabidopsis* significantly inhibits the transcription of *LHY* and *CIRCADIAN CLOCK-ASSOCIATED 1* (*CCA1*), demonstrating that TOC1 can repress the transcription of its target genes *via* binding to their promoters (Gendron et al., 2012). We confirmed that GmTOC1b and AtTOC1 shared the highly conserved CCT domain, which agreed with the notion that GmTOC1b may bind to the promoters of target genes. In this study, we detected two or more TOC1-binding *cis*-elements in the promoter regions of *GmNIN2a* and *GmENOD40-1*. We further confirmed that GmTOC1b can repress their transcription, likely by directly binding to the *GmNIN2a* and *GmENOD40-1* promoters (**Figures 6B–E**).

NINs and ENOD40 play a vital role in rhizobia symbiosis. The expression of their encoding genes is rapidly induced during nodulation, and their corresponding knockout mutants markedly reduced nodule number in both *L. japonicus* (Kumagai et al., 2006; Yoro et al., 2014) and *M. truncatula* (Marsh et al., 2007; Wan et al., 2007). A recent study suggested that light-induced GmSTFs and GmFTs move from shoots to roots, and GmFT2a directly interacts with GmCCaMK-phosphorylated GmSTF3 to form a complex, which directly activates the expression of *NIN* and nuclear factor Y to regulate nodulation (Wang et al., 2021b). Nodule Number Control 1 (GmNNC1) interacts with GmNINa to release the transcriptional repression of *GmENON40 *imposed by NNC1 to regulate nodulation (Wang et al., 2019). *SQUAMOSA PROMOTER BINDING PROTEIN-LIKE 9* (*GmSPL9d*) was co-expressed with *GmNINa* and *GmENOD40-1*, and GmSPL9d positively regulated nodulation by inducing the transcription of *GmNINa* and *GmENOD40-1* (Yun et al., 2022). RT-qPCR analysis of the *Gmtoc1b* mutants and *GmTOC1b-*OE transgenic hairy roots showed that the expression levels of *GmNIN2a*, *GmNIN2b*, and *GmENOD40-1* were negatively regulated by GmTOC1b (**Figures 5A–F**). However, only the transcription of *GmNIN2a* and *GmENOD40-1* was directly repressed by GmTOC1b, while the change in *GmNIN2b* expression could not be explained. Because many genes have been reported to regulate *NIN* expression in soybean, we speculate that GmTOC1b may indirectly regulate *GmNIN2b* expression by directly regulating other nodulation-related genes upstream of *NIN*, which needs to be explored in our future studies.

## Data availability statement

The original contributions presented in the study are included in the article/**Supplementary Material**. Further inquiries can be directed to the corresponding authors.

## Author contributions

YZ performed phenotypic observation and data analysis. QC performed the gene cloning and generation of *Gmtoc1b* mutant. CL and LL performed the subcellular localization. CG and ZC performed the soybean hairy root transformation. YW performed the transient expression assay. BL, FK, and LC wrote the manuscript. All authors contributed to the article and approved the submitted version.

## Funding

This work was supported by the National Natural Science Foundation of China (32001502, 32090064), the Major Program of Guangdong Basic and Applied Research 2019B030302006, and the China Postdoctoral Science Foundation (2020M682655).

## Conflict of interest

The authors declare that the research was conducted in the absence of any commercial or financial relationships that could be construed as a potential conflict of interest.

## Publisher’s note

All claims expressed in this article are solely those of the authors and do not necessarily represent those of their affiliated organizations, or those of the publisher, the editors and the reviewers. Any product that may be evaluated in this article, or claim that may be made by its manufacturer, is not guaranteed or endorsed by the publisher.

## Supplementary material

Supplementary Figure 1 Generation and identification of the *Gmtoc1b* mutants. **(A)** Sequencing verification of CRISPR/Cas9-edited *Gmtoc1b-1* and *Gmtoc1b-2* mutants. **(B)** Relative *GmTOC1b* transcript levels in W82, *Gmtoc1b-1*, and *Gmtoc1b-2* mutants. Three biological replicates were performed in all experiments. All data were normalized to the transcript level of reference gene *GmActin*.

Supplementary Figure 2 Knockout of *GmTOC1b* increases small nodule numbers at 7 DAI. **(A)** Representative phenotype of small nodules observed in W82 and the *Gmtoc1b* mutants at 7 DAI. Scale bars, 100 mm. **(B)** Number of small nodules in W82 and the *Gmtoc1b* mutants at 7 DAI (n = 5).

Supplementary Figure 3 Content of leghemoglobin in nodules from W82 and *Gmtoc1b* mutants. Data are means ± SD of three independent samples. Lowercase letters indicate significant differences as determined by Student’s *t*-test.
