# Supplementary material for: GmTOC1b inhibits nodulation by repressing GmNIN2a and GmENOD40-1 in soybean
Source: Front Plant Sci. 2022 Nov 11;13:1052017. doi: 10.3389/fpls.2022.1052017 (PMC9691777; doi:10.3389/fpls.2022.1052017)
Supplement: Supplementary file 1 [file DataSheet_1.pdf]

Table S1. The specific sequences of the primers.

Primer	Sequence (5'-3')
GmTOC1b-OE-F	TCTAGAATGGAGTCTGGTGAGGAGAT
GmTOC1b-OE-R	GGTACCAGCATCCTCAGGAGAAGAAT
GmTOC1b-GFP-F	TCTAGAATGGAGTCTGGTGAGGAGAT
GmTOC1b-GFP-R	GGCGCGCCAGCATCCTCAGGAGAAGAAT
qGmTOC1b-F	AATCAGCCACCACCGTTTTC
qGmTOC1b-R	CACAACGAACCCTGGACTTG
qGmNIN2a-F	CTTGGCACTTCCTGTCTT
qGmNIN2a-R	ACACCTCATCCTCGTCAA
qGmNIN2b-F	TCTCTCATCCATGATCACCATC
qGmNIN2b-R	ACTGTCCCAGTTGCAGTAGTGG
qGmENOD40-1-F	TCTCTCTTGAGTGGCAGAAGCA
qGmENOD40-1-R	TGGAGTCCATTGCCTTTTCG
qGmActin-F	ACCCAGCAGCATGAAGATCA
qGmActin-R	CACATCTGCTGGAAGGTGCT
GmNIN2apro-F	AAGCTTGATCCGGACTTTGCTAGTGAGT
GmNIN2apro-R	GGATCCGCTACCTGTTCCTTTAATATCC
GmENOD40-1pro-F	AAGCTTGGGGCAACCAAGATTATGGCAA
GmENOD40-1pro-R	GGATCCGCTTCTTCAAGAACCATGGATG
