# Supplementary figures and images for: GmTOC1b inhibits nodulation by repressing GmNIN2a and GmENOD40-1 in soybean
Source: Front Plant Sci. 2022 Nov 11;13:1052017. doi: 10.3389/fpls.2022.1052017 (PMC9691777; doi:10.3389/fpls.2022.1052017)

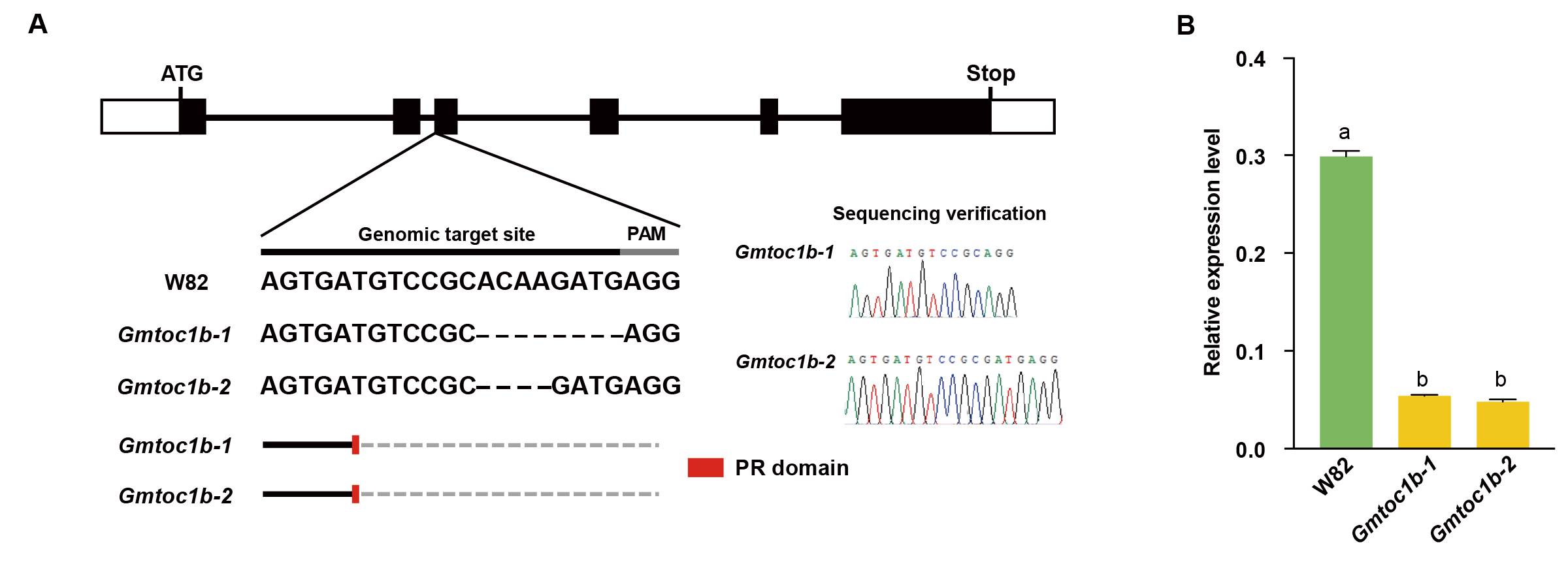

Supplement: Supplementary Figure 1 — Generation and identification of the Gmtoc1b mutants. (A) Sequencing verification of CRISPR/Cas9-edited Gmtoc1b-1 and Gmtoc1b-2 mutants. (B) Relative GmTOC1b transcript levels in W82, Gmtoc1b-1, and Gmtoc1b-2 mutants. Three biological replicates were performed in all experiments. All data were normalized to the transcript level of reference gene GmActin. [file Image_1.jpg]

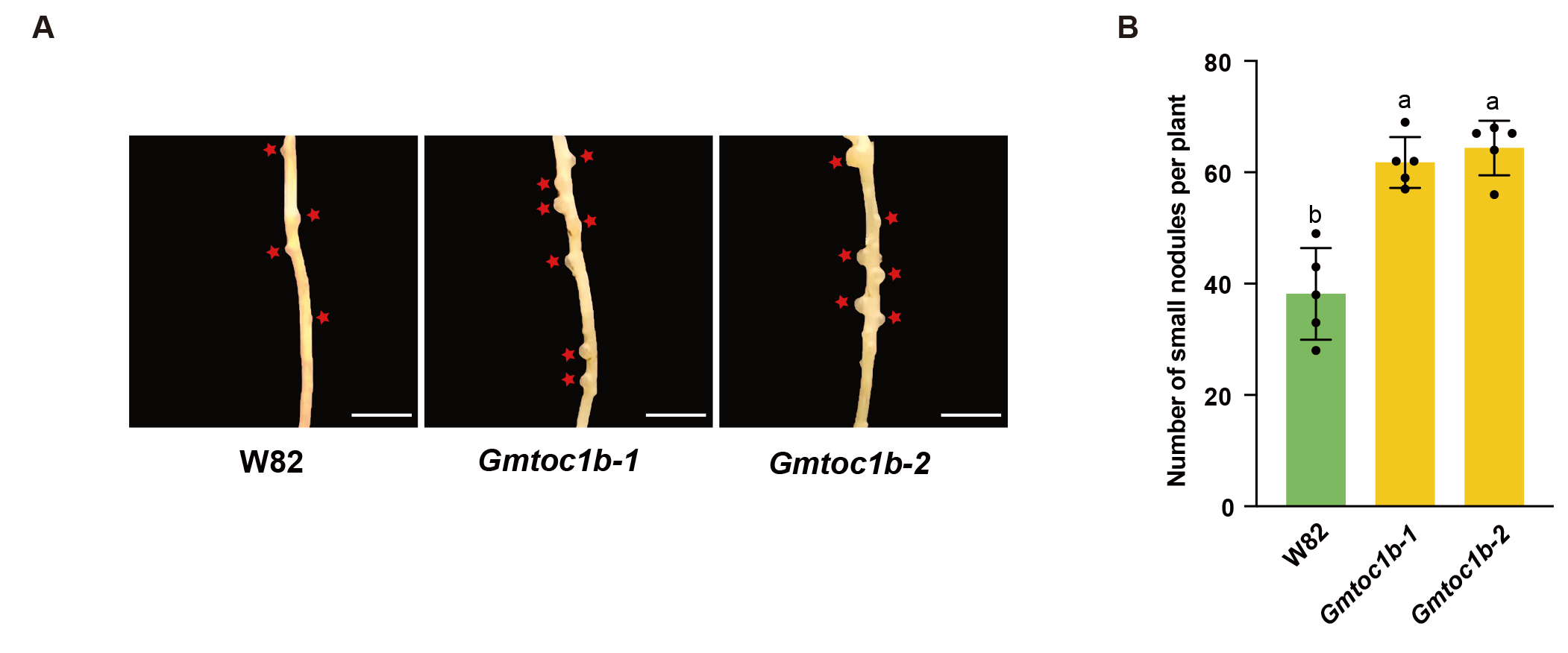

Supplement: Supplementary Figure 2 — Knockout of GmTOC1b increases small nodule numbers at 7 DAI. (A) Representative phenotype of small nodules observed in W82 and the Gmtoc1b mutants at 7 DAI. Scale bars, 100 mm. (B) Number of small nodules in W82 and the Gmtoc1b mutants at 7 DAI (n = 5). [file Image_2.jpeg]

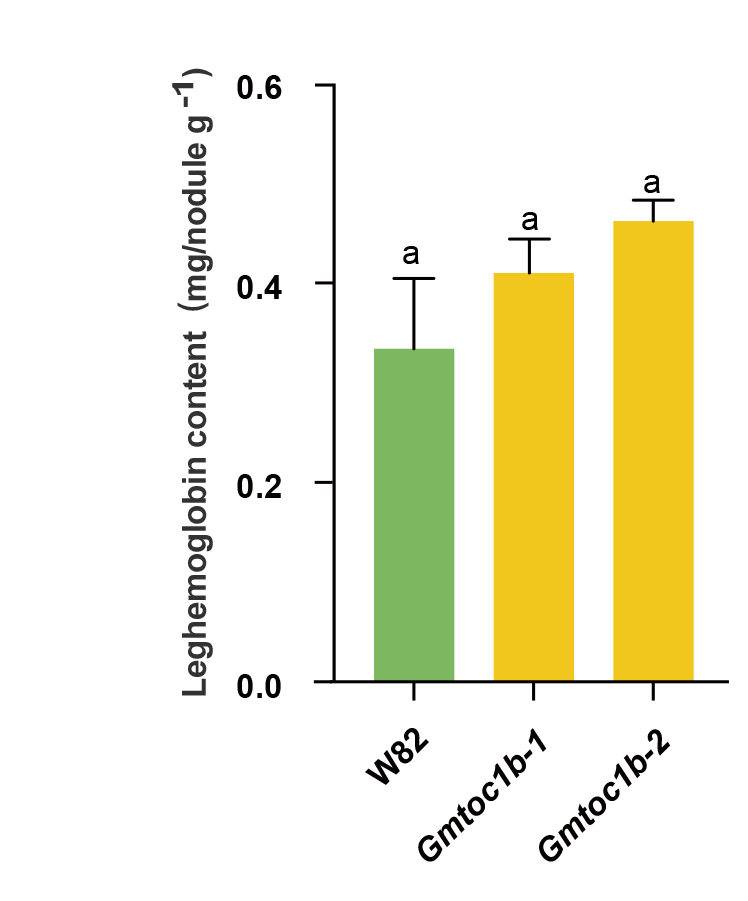

Supplement: Supplementary Figure 3 — Content of leghemoglobin in nodules from W82 and Gmtoc1b mutants. Data are means ± SD of three independent samples. Lowercase letters indicate significant differences as determined by Student’s t-test. [file Image_3.jpeg]
